# Galanin Receptors: G Protein-Dependent Signaling and Beyond

**DOI:** 10.3390/biom16020236

**Published:** 2026-02-03

**Authors:** Judit Oláh, Eszter Soltész-Katona, Hana Kaci, Gábor Turu, László Hunyady

**Affiliations:** 1 Institute of Molecular Life Sciences, Centre of Excellence of the Hungarian Academy of Sciences, HUN-REN Research Centre for Natural Sciences, 1117 Budapest, Hungary; soltesz-katona.eszter@ttk.hun-ren.hu (E.S.-K.); kaci.hana@ttk.hun-ren.hu (H.K.); turu.gabor@ttk.hun-ren.hu (G.T.); hunyady.laszlo@ttk.hun-ren.hu (L.H.); 2 Department of Physiology, Semmelweis University, 1094 Budapest, Hungary

**Keywords:** galanin receptor, β-arrestin, receptor structure, biased signaling, pathological function

## Abstract

The G protein-coupled galanin receptors include three different subtypes: galanin receptor 1, 2 and 3 (GalR1, GalR2, GalR3). The neuropeptide galanin is the principal natural agonist of the galanin receptors, the so-called galaninergic system. Galanin-like peptide and spexin have also been identified as natural ligands of the galanin receptors. Galanin receptors are widely expressed in the brain; however, they can be found in other tissues, such as the skeletal muscle, the heart, and the gastrointestinal tract. The galaninergic system regulates diverse biological processes, including feeding behavior, neuroprotection, learning, memory, cardiovascular and renal function, and nociception. Its dysregulation is associated with various diseases, such as Alzheimer’s disease, diabetes mellitus, epilepsy, depression, and cancer. The stimulation of GalR1 and GalR3 leads to the Gαi/o-type G protein-mediated inhibition of cyclic AMP/protein kinase A, whereas GalR2 stimulation initiates phospholipase C activation via Gαq/11-type G proteins. A galanin-activated β-arrestin-dependent pathway has also been described for GalR2. In this review, we summarize the recent advances concerning galanin receptor signaling, including both the G protein-dependent and -independent pathways. A better understanding of the complex interplay of the signaling molecules, receptors, and various signaling pathways is crucial for the future development of specific agonists with therapeutic potential.

## 1. Introduction: The Galaninergic System

**Figure 1:**
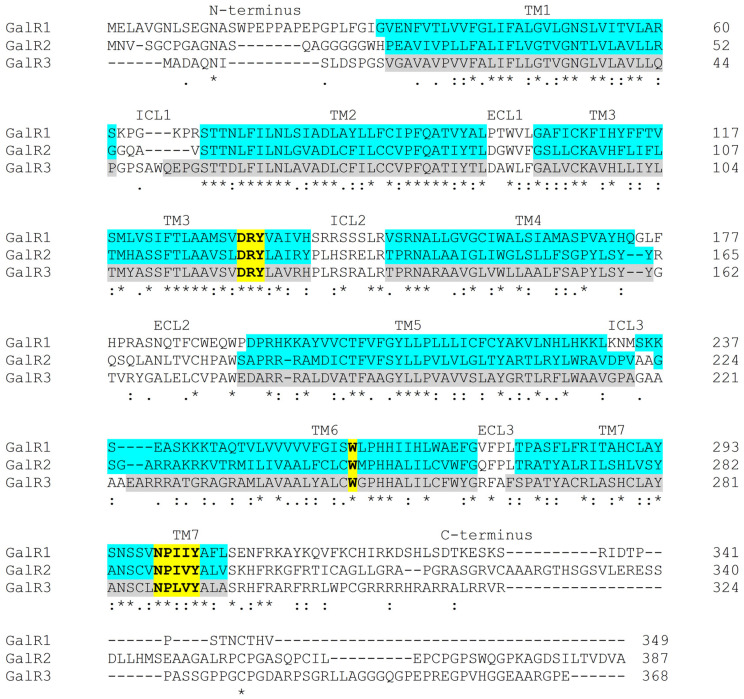
Sequence comparison of the three human galanin receptor subtypes (GalR1, GalR2 and GalR3) by Clustal Omega (version 1.2.4) [8]. Identical and similar amino acid residues are indicated by asterisks and colons/dots, respectively. The transmembrane helices (TM) are highlighted in light blue for GalR1 and GalR2 (based on the determined structures [9,10,11], https://gpcrdb.org/protein/galr1_human/ and https://gpcrdb.org/protein/galr2_human/, accessed on 7 December 2025) and in grey for GalR3 (using https://gpcrdb.org/protein/galr3_human/, accessed on 7 December 2025). The highly conserved Trp toggle switch in TM6, the Tyr toggle switch (NPxxY motif in TM7) and the DRY motif (in TM3) are highlighted in yellow (bold) [12]. Intracellular loop, ICL; extracellular loop, ECL.

G protein-coupled receptors (GPCRs) are the largest family of signaling proteins; they are among the most common therapeutic targets [1]. Most classification systems of GPCRs are based on their structural similarity. Galanin receptors are identified as class A GPCRs [2,3]. The galanin receptors include three different subtypes in humans: galanin receptor 1, 2 and 3 (GalR1, GalR2, GalR3, respectively) [4,5,6] (Figure 1). All GPCRs share a highly conserved structure composed of an extracellular N-terminus, seven transmembrane α-helices (TMs), three intracellular loops (ICLs), three extracellular loops (ECLs), and an intracellular C-terminus [7]. The homology among the galanin receptors is only 36–54% [3]; the transmembrane helices show the highest similarity, whereas the terminal regions differ substantially.

The principal endogenous agonist of the galaninergic system is galanin, a 30-amino acid neuropeptide in humans, which was discovered in the early 1980s [13]. After the cloning of *Gal* gene, a further peptide product was found to be encoded by it, the galanin message-associated peptide (GMAP), which is 61-amino acid long in humans [14]. Later, other endogenous peptides were found such as galanin-like peptide (GALP) [15], alarin [16], and more recently, spexin [17]. A second form of spexin, SPX2, was identified in non-mammalian species, but this form was not detected in humans [17].

These peptides are derived from a prepro-protein, for example, the proteolytic cleavages of *Gal* result in the galanin and GMAP. The N-terminus of galanin, the first 19 amino acids, is highly conserved throughout evolution, while its C-terminal part is much less conserved. The 60-amino acid GALP is derived from another prepro-peptide; however, its 9-21-amino acid region is identical to the first 13 amino acids of galanin. A further transcript is derived from GALP, the 25-amino acid-long alarin, but due to alternative splicing, its sequence is different. It is also considered part of the galaninergic system; nevertheless, it does not bind to galanin receptors. So far, no receptor is known for either alarin or GMAP. It has also been suggested that although GALP binds to galanin receptors, it may regulate its actions through yet unknown receptors instead of GalR1-3 [18].

Several aspects of the galaninergic system have been reviewed since their discovery, especially the characterization of their G protein-dependent signaling pathways and their roles in various physiological and pathological processes. A milestone in the research field has been the recent determination of galanin receptor structures by cryo-electron microscopy (cryo-EM) [9,10,11]. The purpose of this review is to summarize the recent advances concerning galanin receptor signaling, including their G protein and β-arrestin-dependent pathways, and the progress in the structural insight into their signaling, and outline some future open questions and challenges.

## 2. The Distribution of the Galanin Receptors and Peptides in Humans

**Figure 2:**
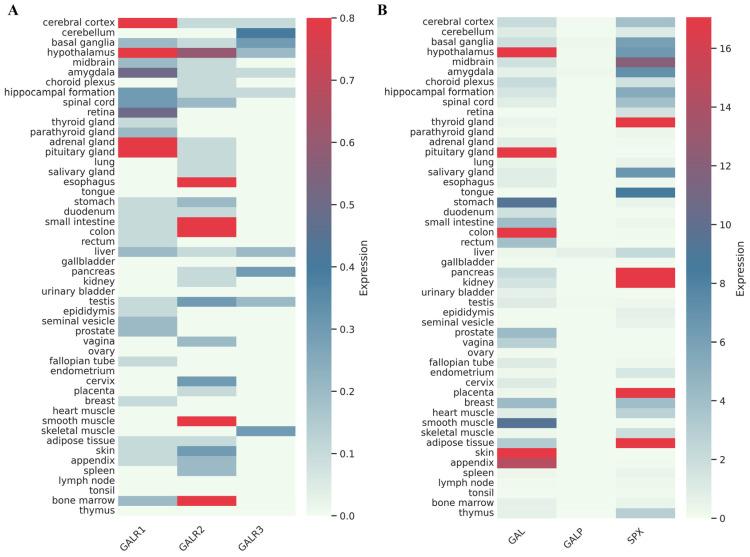
Distribution of human galanin receptors (**A**) and their agonists (**B**), nTPM values from the Human Protein Atlas (https://www.proteinatlas.org/ENSG00000166573-GALR1; https://www.proteinatlas.org/ENSG00000182687-GALR2; https://www.proteinatlas.org/ENSG00000128310-GALR3; https://www.proteinatlas.org/ENSG00000069482-GAL; https://www.proteinatlas.org/ENSG00000197487-GALP; https://www.proteinatlas.org/ENSG00000134548-SPX, all websites accessed on 21 November 2025).

The distribution of galanin and other peptides’ mRNA, as well as that of the galanin receptors, has been characterized in various species and reviewed in detail (see [3] and references therein). However, significant differences were observed even among primates let alone among mammals [19]. Therefore, normalized gene expression values taken from the Human Protein Atlas were used to represent the distribution of the galanin receptor mRNA, as well as that of the endogenous agonists (Figure 2). These values are reported as transcripts per million (nTPM) units. Data concerning the protein levels often cannot be validated (for example, in the case of GalR1 and GalR3) due to the questionable specificity of the available antibodies. For GalR2, both the RNA and the protein expression data are available; however, the consistency between antibody staining and RNA expression is rather low.

The highest GalR1 mRNA levels can be found in endocrine tissues and various parts of the brain, such as cerebral cortex, hypothalamus, and amygdala (Figure 2). In contrast, the expressions of GalR2 and GalR3 are more widespread; they have been observed in peripheral tissues as well (Figure 2). GalR2 mRNA is highly expressed in the gastrointestinal tract, muscle tissue, and bone marrow, although it can also be detected in the hypothalamus. GalR3 mRNA was detected in various brain areas, and its expression is also relatively high in the pancreas, skeletal muscle, and liver (Figure 2).

The localization of galanin and the three subtypes of galanin receptors was studied in the locus coeruleus (LC) and the dorsal raphe nucleus (DRN) in human postmortem brain [20]. The LC is of special interest, since it plays crucial roles in the modulation of several behavioral and physiological processes, as well as in depression, anxiety, and neurodegeneration under pathological conditions [21,22]. The LC consists of predominantly noradrenergic neurons, while the DRN comprises mainly 5-hydroxytryptamine (5-HT; serotonin) neurons. In this study, galanin and GalR3 mRNA were detected in many noradrenergic LC neurons [20]. An overlap between GalR3 and serotonin neurons was also detected in the DRN, but galanin was not detected in 5-HT-DRN neurons. The major galanin receptor in these neurons is GalR3, while GalR2 and GalR1 are expressed at low levels, if at all. However, in the forebrain, the most abundant expression was found for GalR1, while GalR3 was likely not expressed [20]. Other studies have also found that galanin is highly expressed in the LC in norepinephrine neurons, and it is expressed in some GABAergic neurons as well [22,23].

In the Human Protein Atlas, the highest galanin mRNA levels can be found in the pituitary gland, followed by those in the hypothalamus, skin, colon, and appendix (Figure 2). Protein expression data are also available for galanin in the Human Protein Atlas. Galanin is mainly expressed in the pituitary gland, hypothalamus, adrenal gland, and the gastrointestinal tract at protein level, indicating a broad agreement between transcriptomic and protein-level datasets at the tissue level. However, for neuropeptides such as galanin, protein localization may extend beyond sites of mRNA expression due to axonal transport and vesicular storage. An early study was carried out to investigate the distribution of galanin-like immunoreactivity in the human brain [24]. The immunoreactive cells were mainly restricted to the basal nucleus of Meynert and to various areas of the hypothalamus; a more widespread fiber staining was observed in the hypothalamus, the diagonal band, the septum, amygdala, hippocampus, and cortex. The authors noted that galanin seems to have a relatively widespread distribution in the human brain, although not as extensive as that described for the rat brain [24].

The highest spexin mRNA levels were observed in adipose tissue, kidney, pancreas, thyroid gland, and placenta. Its expression was studied in human tissues, and higher expression was found in several tissues (adrenal gland, skin, stomach, small intestine, liver, thyroid, pancreatic islets, visceral fat, lung, colon, and kidney) as compared to expression in muscle and connective tissues [25]. Spexin is also widely expressed in the brain, including the hypothalamus [26]. GALP is expressed mainly in the hypothalamic arcuate nucleus and the posterior pituitary [27].

## 3. Structural Insight into the Ligand Binding and Specificity of Galanin Receptors

Class A (rhodopsin family) GPCRs are characterized by a relatively short N-terminal extracellular domain, their binding pocket is usually deep and narrow [28,29]. A common binding pocket involving residues of TM2, TM3, TM6, TM7 and ECL2 was suggested for the peptide agonists [29]. In class A GPCRs, agonist binding induces several conformational changes [30,31]. Upon activation, the rotation and outward displacement of TM6 accompanied by inward movement of TM5 and TM7 as well as the rotation and upward movement of TM3 lead to the formation of a cavity, this intracellular surface can interact with the various transducers, such as G proteins and β-arrestins. The conformational changes stem from the rearrangement of the hydrophobic receptor core and characteristics motifs, such as the toggle switch Trp^6.48^ (in the Cys/Ser/Thr^6.47^-Trp^6.48^-Phe^6.50^ motif), the DRY (Glu/Asp^3.49^-Arg^3.50^-Tyr/Trp^3.51^) and the NPxxY (Asn^7.49^-Pro^7.50^-XX-Tyr^7.53^) motifs (superscripts denote Ballesteros–Weinstein numbering) [30,31,32].

**Figure 3:**
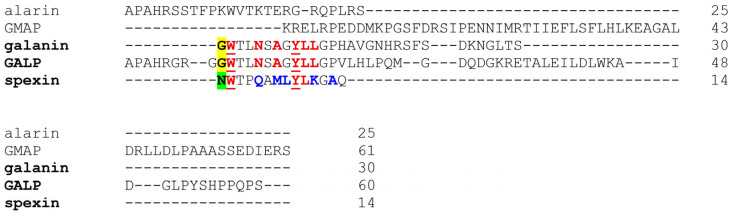
Sequence comparison of the peptide agonists in the human galaninergic system by Clustal Omega (version 1.2.4) [8]. Critical residues of endogenous agonists in galanin receptor binding as determined by alanine scanning mutagenesis and cryo-EM are shown in red. The first amino acid residue of galanin/GALP and spexin is highlighted in yellow and green, respectively. Residues of spexin involved in the ligand selectivity are shown in blue.

After the discovery of galanin receptors, alanine scanning mutagenesis studies gave insight into the structural aspects of their ligand binding [33,34]. The N-terminal part of galanin is highly conserved across species, and the binding affinity of galanin (1–16) is comparable to that of the full-length agonist. Trp2, Asn5, Tyr9, Leu10, Leu11, and the free N-terminal amino group of galanin were found to be important pharmacophores, and its Trp2 and Tyr9 are especially critical for ligand binding [33,35] (Figure 3). Early studies identified important residues involved in the formation of the binding pocket of GalR1, such as His264^6.52^ and Phe282^7.32^, which interact with Tyr9 and Trp2 of galanin, respectively [33,34]. An important difference between GalR1 and GalR2 is that, although both bind galanin with high affinity, only GalR2 displays high affinity for the N-terminally truncated galanin (2–30) form.

An interesting aspect of the galanin receptor subtypes is ligand selectivity. All three receptors bind galanin, which displays high affinity for GalR1 and GalR2, but lower affinity for GalR3 [17], meanwhile only GalR2 and GalR3 can be stimulated by spexin. There is a synthetic, spexin-based GalR2 agonist, SG2A. Similarly to spexin, SG2A induces GalR2 signaling via Gαq protein, but it does not stimulate GalR3. Using site-directed mutagenesis and domain swapping between GalR2 and GalR3, Lee and co-workers provided an explanation for the ligand selectivity of SG2A [36]. When four residues in spexin were substituted with residues derived from the corresponding positions in galanin (Asn5, Ala7, Leu11/Phe11, and Pro13), this quadruple mutant displayed full GalR2 specificity. They also identified specific amino acids within TM3, TM5, and ECL3 of GalR2 that promote favorable interaction with SG2A as well as residues in GalR3 that inhibit the interaction [36].

More detailed studies have been carried out only recently, and three publications have been published almost at the same time. The receptor structures determined by cryo-EM are available for GalR1 (galanin-bound structures 7WQ3 [11], 7XJJ [9]) and GalR2 (galanin-bound structures 7XBD [10], 7WQ4 [11], 7XJK [9]; spexin-bound receptor 7XJL [9]) in the Protein Data Bank [9,10,11]. These studies provide insight into ligand binding, receptor subtype, and ligand selectivity.

**Figure 4:**
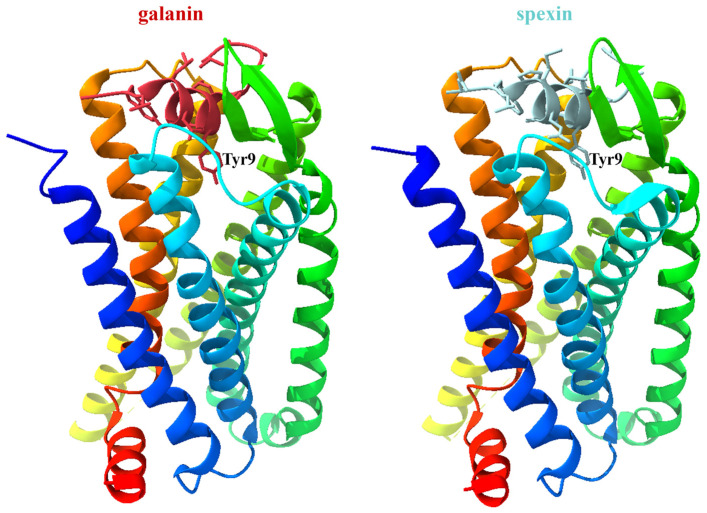
Structure of galanin- and spexin-bound GalR2 (7XJK and 7XJL) [9]. Galanin and spexin are highlighted in red and light blue, respectively. Tyr9 of the agonists is labeled. The GalR2 receptor is displayed in cartoon representation and color-coded from the N- to the C-terminus (blue to red) to facilitate identification of transmembrane helices and extracellular and intracellular loop regions.

Duan and co-workers determined the receptor structures of GalR1 (7WQ3) and GalR2 (7WQ4) in complex with galanin and their primary G protein subtypes, Gαi and Gαq, respectively [11]. It was found that galanin displays a unique binding mode, as it almost “lays flat” on top of the receptor transmembrane domain pocket. The N-terminal part of galanin (Leu4-Leu11) adopts an α-helical conformation, which differs from the loop and β-strand conformations characteristics of other peptide agonists such as angiotensin II (AngII), the agonist of the type 1 angiotensin II receptor (AT_1_R). In contrast to the majority of other peptide ligands, which penetrate into the transmembrane helical domain (TMD) binding cavity, Tyr9 is the only residue of galanin inserted into the cavity (Figure 4). However, the receptor cavity has a similar depth to that found in other receptors, and there is extra space in the binding pocket below Tyr9 of galanin [11].

Hydrophobic residues localized in ECL2, TM6, ECL3, and TM7 interact with galanin. The N-terminal helix of galanin is sandwiched by two hydrophobic patches, causing the agonist “lay flat” at the extracellular surface. Trp2, Ala7, Leu10, and Leu11 of galanin face a similar hydrophobic patch formed by TM6, ECL3, and TM7 in both receptor subtypes. Leu4 of galanin is in contact with aromatic residues of ECL2 in both GalR1 and GalR2; Pro13 and Val16 of galanin also interact with these residues in GalR1, but not in GalR2. The contribution of ECL2 to galanin binding is greater in GalR1 than in GalR2. This may explain the previous observation that GALP, whose residues 9–21 are identical to residues 1–13 of galanin, displays a higher affinity for GalR2 than for GalR1.

The comparison of the structures also revealed that Gly1 of galanin forms an H-bond with Glu32^1.31^ in GalR1, while there was no significant interaction between Gly1 of galanin and residues of GalR2, which explains the receptor subtype selectivity for galanin (2–16) [11]. Tyr9 is the same residue in both galanin and spexin, but their first residues differ—Gly1 in galanin and Asn1 in spexin, respectively. The fact that spexin does not activate GalR1 further highlights the importance of Gly1 in GalR1 activation.

Upon activation, both subtypes of galanin receptors display a pronounced outward displacement of the cytoplasmic end of TM6, which is characteristic of class A GPCRs activation, coupled with the movement of TM7. Several positively charged residues were identified in TM6 and TM7 below the galanin binding site, and the authors proposed that these residues may connect the peptide pocket to the toggle switch [11]. The critical role of ICL2 in Gαq selectivity has also been revealed [11]. GalR1 lacks a conserved hydrophobic residue in ICL2, which is often involved in association with the Gα subunit; ICL2 of GalR1 is not involved in the interaction with Gαi. In contrast, Leu131^34.51^ occupies this position in ICL2 of GalR2, which forms a crucial hydrophobic interaction with Gαq [11]. It was further confirmed when ICL2 of GalR1 was replaced with that of GalR2, the chimera receptor showed Gαq-mediated signaling [11].

In another study, GalR1 (7XJJ) and GalR2 (7XJK) were fused to a mini-Gαo and a mini-Gαq protein, respectively, for the determination of the receptor structure [9]. The galanin peptide predominantly adopts a helical structure, which binds to the extracellular vestibule of the receptor. However, it is nearly parallel to the membrane plane and does not penetrate deeply into the receptor core. The peptide is distant from the toggle switch (Trp260^6.48^ in GalR1 and Trp249^6.48^ in GalR2, respectively), the conformational change in which is critical for receptor activation [9]. This was surprising, since most neuropeptides of class A GPCRs bind nearly perpendicular to the membrane plane, and these peptides penetrate the helical cavity close to the toggle switch. Galanin was found to be in contact with all seven TMs and ECL2 and ECL3, and it occupies a significant surface area, which explains its high affinity to the receptors. Tyr9 of galanin was essential for the binding, as this residue penetrates into the receptor core (Figure 4). The residues of GalR1 and GalR2 involved in galanin binding are distinct, although there is some overlap. Gly1 of galanin lies between TM1 and TM7 but is positioned closer to TM1 in GalR1 than in GalR2, which accounts for its greater contribution to GalR1 binding affinity [9].

ECL2 and ECL3 are also involved in galanin binding. ECL2 forms an antiparallel β-sheet and covers galanin as a lid-like structure through hydrophobic interactions. Distinct residues of ECL2 (Trp188^45.51^ and Phe186 of GalR1; His176^45.51^ and Val174 of GalR2) and ECL3 (Val274 of GalR1, Gln263 of GalR2) can be found in GalR1 and GalR2, and the amino acids of ECL2 in GalR1 have bulkier aromatic side chains than in GalR2. Spexin is an endogenous agonist for GalR2 and GalR3, but not for GalR1. While in galanin Ala7 and Gly8 precede Tyr9, Met7 and Leu8 are present in spexin. The spexin-bound GalR2 structure (7XJL) provided an explanation for the specificity of spexin, because Leu8 may clash with the bulkier residues in GalR1 [9].

An α-helical structure is characteristic of the ICL2 of most Gαi/o-coupled receptors, but the ICL2 in GalR1 is disordered. Substitution of critical amino acids of ICL2 of GalR1 and GalR2 with each other revealed that the alteration significantly reduced the association of GalR2 and Gαq but had little effect on the ability of GalR1 to couple to Gαi [9]. Based on data from various mutants, the ICL2 region of GalR2, specifically Pro130 and Leu131, and the ICL3 of GalR1 contribute to the selectivity toward Gαq and Gαo, respectively [9].

In another study, a complex composed of GalR2, galanin, and mGαq_iN_/Gβ1γ2 was used to determine the structure (7XBD) [10]. TM5–7 of GalR2 forms a hydrophobic pocket that accommodates mGαq_iN_. A relatively shallow galanin binding pocket was formed by the TM helices of GalR2 at the extracellular part of the receptor. Galanin is in contact with each ECL and also binds to hydrophobic residues near the N-termini of TM2 and TM7. The authors also found that Trp2, Asn5, Tyr9, and Leu10 residues of galanin are critical for receptor binding [10]. Tyr9 of galanin interacts with Ile85^2.64^, His102^3.29^ and Tyr164^4.64^ of GalR2, while His176^45.51^ (in ECL2 of GalR2) was found to be in contact with Asn5 of galanin. His176^45.51^ displays a modest contribution to agonist binding. This residue is unique to GalR2; Trp and Val are found in this position in GalR1 and GalR3, respectively. The authors raised the possibility that these distinct residues may explain the different strengths of galanin binding to the receptors [10].

Although the above studies used slightly different complexes for the determination of the structure, the main conclusions were similar and provided an explanation for several characteristics of galanin receptors. In summary, these structural studies revealed a unique binding mode for galanin, as galanin is nearly parallel to the membrane plane. Two conserved hydrophobic patches formed by TM6, TM7 and all three ECLs of GalR1/GalR2 interact with galanin resulting in a large extracellular crevice. The N-terminus of galanin is sandwiched by the two patches and almost “lay flat” at the extracellular surface of the receptor, only Tyr9 of galanin penetrates to some extent into the receptor core [9,11]. In contrast, endogenous agonists for other class A GPCRs bind perpendicular to the membrane plane and usually penetrate into the receptor core by their extreme N-terminus (such as [D-Ala2, NMe-Phe4, Gly-ol5]-enkephalin (DAMGO), a μ-opioid receptor (μOR) agonist), C-terminus (AngII) or cyclic middle segment (arginine-vasopressin (AVP)) [11]. Comparison of galanin-bound structures of GalR1 and GalR2, as well as that of galanin- and spexin-bound GalR2 provided an explanation for ligand specificity and the critical role of Gly1 of galanin in GalR1 binding. These structural studies also revealed that ICL2 of GalR2 and ICL3 of GalR1 may determine the selectivity of receptors toward Gαq and Gαo, respectively. To our best knowledge, no structure has been determined yet for GalR3 by cryo-EM.

## 4. G Protein and β-Arrestin-Coupled Signaling Pathways of Galanin Receptors

### 4.1. G Protein-Dependent Signaling

Differences in agonist preference across galanin receptor subtypes translate into distinct G protein-dependent signaling profiles (Table 1) [17]. The activation of GalR1 by galanin results in the inhibition of adenylate cyclase, coupled with decreased cyclic adenosine 3,5-monophosphate (cAMP) levels, opening/activation of the G protein–gated inwardly rectifying potassium channels (GIRKs), and stimulation of mitogen-activated protein kinase (MAPK) activity [4,37,38,39]. These processes are mediated by Gαi/o-type G proteins [40]. Stimulation of the MAPK pathway through Gαo-type G protein was described for GalR2. Nevertheless, its signaling is predominantly mediated through Gαq/11-type G protein resulting in phospholipase C activation and increased Ca^2+^ level, GalR2 stimulates large-conductance Ca^2+^-activated K^+^ channels (BKs) [40,41]. Signaling via Gα12/13-type G proteins was also proposed for GalR2, with the subsequent activation of Rho A protein [42]. GalR3 is less characterized, and seems to be similar to GalR1, because GalR3 signaling is also mediated by Gαi/o-type G proteins resulting in the inhibition of adenylate cyclase and the reduction in cAMP concentration [37]. One of the main challenges in studying GalR3 is that it is poorly expressed at the plasma membrane in recombinant systems [43]. In transiently transfected cells (HEK293, CHO, or PC12), primarily intracellular staining could be detected, and only after permeabilization. It was suggested that the C-terminal tail of GalR3 contains putative endoplasmic reticulum retention motifs [43].

The stimulation of GalR2 and GalR3 by spexin initiated the phosphatidylinositol 3-kinases (PI3K)/protein kinase B (PKB) and cAMP/protein kinase A (PKA) signaling pathways [44]. Spexin also upregulates the extracellular signal-regulated kinase1/2 (ERK1/2) pathway and activates the L-type voltage-dependent Ca^2+^ channel (VDCC) [44,45]. A galanin-galanin receptor signaling network was constructed, integrating the known interactions into a freely available map in 2021 (https://www.wikipathways.org/index.php/Pathway:WP4970, accessed on 7 December 2025) [46].

**Table 1:** Characteristics of the galanin receptors.

		GalR1 P47211	GalR2 O43603	GalR3 O60755
Endogenous agonists	galanin P22466	pKi 9.1–10.5 [4,47]	pKi 8.6–9.1 [48,49]	pKi 7.2 [37]
			pEC50 7.1 [17]	pEC50 5.9 [17]
	spexin Q9BT56		pEC50 6.8 [17]	pEC50 6.2 [17]
	GALP Q9UBC7	pIC50 7.1 [50]	pIC50 7.6–7.7 [15,50]	pIC50 8.0 [50]
	alarin	pIC50 < 6.0 in rat [51]	in rat pIC50 < 6.0 [51]	in rat pIC50 < 6.0 [51]
G protein-dependent pathway		Gαi/o-type G proteins ↓ adenylate cyclase ↓ cAMP ↑ MAPK activity ↑ GIRK (GIRK1, GIRK2, GIRK4) [38,39,40]	Gαq/11-type G proteins ↑ phospholipase C ↑ Ca^2+^-dependent ion channels (BK), ↑ Ca^2+^ [40,41]	Gαi/o-type G proteins ↓ adenylate cyclase ↓ cAMP ↑ GIRK (GIRK1, GIRK4) [37,44]
			Gαo-type G proteins ↑ MAPK activity [40]	
			Gα12/13-type G protein ↑ Rho A [42]	
			spexin: ↑ cAMP/PKA ↑ PI3K/PKB, ↑ ERK1/2 ↑ VDCC [44,45]	spexin: ↑ cAMP/PKA ↑ PI3K/PKB, ↑ ERK1/2 ↑ VDCC [44,45]
β-arrestin		yes [52]	yes [53]	?
		internalization [54,55]	internalization [56]	?

Uniprot identifiers are shown for the receptors and the agonists. ↓: decrease, ↑: increase, ?: not known. Abbreviations: cyclic adenosine 3,5-monophosphate (cAMP); extracellular signal-regulated kinase1/2 (ERK1/2); galanin-like peptide (GALP); G protein–gated inwardly rectifying potassium channel, GIRK; large-conductance Ca^2+^-activated K^+^ channel (BK); L-type voltage-dependent Ca^2+^ channel (VDCC); mitogen-activated protein kinase (MAPK); phosphatidylinositol 3-kinases (PI3K); protein kinase A (PKA); protein kinase B (PKB).

### 4.2. β-Arrestin-Dependent Signaling of GPCRs

Ligand binding to GPCRs initiates the canonical heterotrimeric G protein-coupled signaling pathways via various second messengers, after which the receptor C-terminus and/or intracellular loops can be phosphorylated by G protein-coupled receptor kinases (GRKs), followed by the binding of β-arrestins (β-arrestin1 and β-arrestin2) to the phosphorylated residues [1,7,57]. This process leads to desensitization and internalization of the receptor, followed by either receptor recycling or its degradation by the lysosomes. β-Arrestins, as scaffolding and regulatory proteins, play crucial roles in these processes, since they can recruit the endocytic machinery for internalization and are also involved in β-arrestin-dependent signaling processes [58]. β-Arrestins, as adaptor molecules, can promote the association of various signaling proteins such as protein phosphatase 2A, MAPKs, and phosphoinositide 3-kinase; β-arrestin signaling can also initiate transcriptional regulatory processes [59].

It has been established that GPCRs differ in the duration and stability of their β-arrestin interactions, allowing their classification into class A and class B. Concerning beta-arrestin interaction, class A receptors (such as the V_1_A vasopressin receptor, β_2_-adrenergic receptor (β_2_AR), D_1_A dopamine receptor, and CB_1_ cannabinoid receptor) exhibit transient, low-affinity β-arrestin binding that dissociates before endosomal trafficking. In contrast, class B receptors (such as AT_1_R, V_2_ vasopressin receptor (V_2_R), and the oxytocin receptor) display stable β-arrestin association, promoting sustained arrestin-dependent signaling [60]. Although β-arrestin1 and β-arrestin2 display high sequence and structural similarities, they interact differently with GPCRs. Receptors with less stable β-arrestin binding (class A receptors such as β_2_AR) seem to prefer β-arrestin2, receptors with stable association (class B receptors such as AT_1_R) display no selectivity [61]. β-arrestins were found to adopt different conformations upon binding to the same GPCR, the active parathyroid hormone 1 receptor, due to differences in their C-domain (C-edge, loop 2), β-arrestin2 preferably stabilized the core conformation of the receptor [62].

Various GPCR agonists can selectively activate particular signaling pathways rather than the general G protein–mediated signaling [63]. This phenomenon, known as “biased agonism” or “functional selectivity,” results in distinct physiological effects. This mechanism was traditionally thought to arise from ligand-specific alterations in GPCR conformation [64], and the classical view held that ligand efficacy reflected differential recruitment of downstream effectors, such as β-arrestins or G proteins. Biased agonism can also be detected with endogenous ligands. AngII-derived peptides can activate AT_1_R with differing affinities and distinct functional selectivity. Ang-(1–7) induces β-arrestin binding without measurable Gαq activation, whereas AngIV, though has lower affinity than AngII, selectively activates the Gαq pathway [65]. Multiple mechanisms may account for biased agonism, such as biased receptor conformations, alterations in signaling kinetics, or subcellular location. We discussed these diverse mechanisms of biased agonism in detail in one of our recent review articles [66].

### 4.3. Structural Insight into β-Arrestin Binding of GPCRs

Considerably fewer structures have been determined for GPRCs in complex with β-arrestins than in complex with G proteins. The structures solved so far provide crucial insight into β-arrestin binding. Their binding to active, phosphorylated GPCRs is thought to involve two types of interaction between a receptor and a β-arrestin [57]. First, the phosphorylated carboxyl terminus (also referred to as the receptor ‘tail’) or ICL3 interacts with Lys residues of the β-strand I in the N-domain of β-arrestins. Then the cytoplasmic surface of the receptor (also referred to as the receptor ‘core’) can also bind β-arrestins through various regions, mainly the so called fingerloop in the β-arrestins, which connects β-strand V and VI [57]. The β-arrestin-biased ligands promoted only the tail interaction (for review see [67]). The exact patterns of phosphorylated Ser/Thr residues, the phosphorylation barcodes, responsible for stable β-arrestin binding are still not well understood. Recently, a machine-learning-based approach was developed to identify such Ser/Thr-rich pattern, which was termed arreSTick [68].

One of the most studied model system for β-arrestin binding is the V_2_P receptor, because of the long-lasting, stable interaction between the receptor and β-arrestin [69,70]. A specific pattern of phosphorylated residues in the C-terminal tail of the receptor binds to the Lys residues in the N lobe of β-arrestins, which ensures their strong interaction. The structure of V_2_R in complex with AVP and β-arrestin1 has been determined by cryo-EM (PDB ID 7R0C) [69]. The conformation of the agonist AVP was similar to those observed in the active AVP-V_2_P-Gαs complexes (such as PDB ID 7BB7). Concerning β-arrestin1, both the rotation of the C-lobe relative to the N-lobe and the movements of the various loops forming a central crest for receptor coupling were detected, which hallmark the active conformation. The C-terminal tail of the receptor was in contact with the N-domain of β-arrestin1, while all ICLs and the 7TM cavity engaged the β-arrestin1 in a core conformation [69].

Recent cryo-EM structures of the μOR bound to Gαz (PDB ID 9WST and 9WSW) and β-arrestin1 (PDB ID 9WSV and 9WSX) reveal key aspects of receptor signaling plasticity [71]. In these complexes, DAMGO and endomorphin1 adopted largely similar binding poses with deep N-terminal insertion into the TDM cavity. The study revealed the key roles of TM1 and helix8 in regulating signaling bias. Comparing Gαi- (PDB ID 8EFQ, [72]) and β-arrestin1-bound forms of the receptor, upward displacement of helix8, outward movement of the TM7–helix8 hinge, and inward displacement of TM1 were observed. While outward TM1 displacement induces G protein recruitment, its inward TM1 retraction stabilizes the intracellular binding pocket (TM2-TM7-helix8 interface) and promotes the β-arrestin1 association [71].

### 4.4. β-Arrestin-Dependent Pathways of the Galanin Receptors

**Figure 5:**
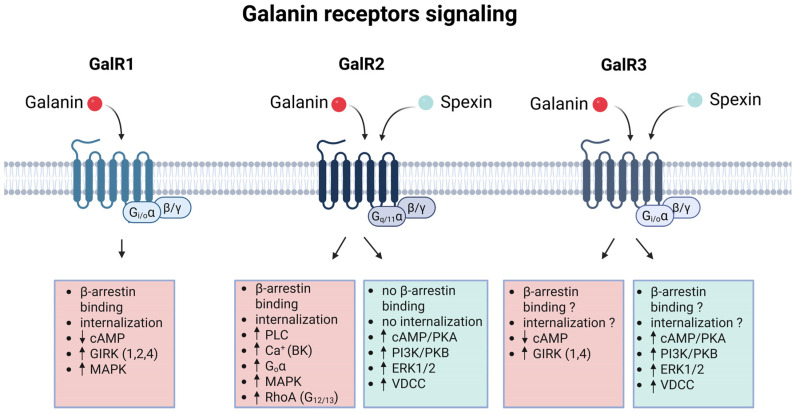
Signaling pathways for galanin receptors. ↓: decrease, ↑: increase, ?: not known. Abbreviations: cyclic adenosine 3,5-monophosphate (cAMP); extracellular signal-regulated kinase1/2 (ERK1/2); G protein–gated inwardly rectifying potassium channel, GIRK; large-conductance Ca^2+^-activated K^+^ channel (BK); L-type voltage-dependent Ca^2+^ channel (VDCC); mitogen-activated protein kinase (MAPK); phosphatidylinositol 3-kinases (PI3K); protein kinase A (PKA); protein kinase B (PKB). Created in BioRender. Turu, G. (2026) https://BioRender.com/8fpgv12.

Concerning galanin receptors, there are only a few studies on their interactions with β-arrestins so far (Figure 5). It was observed relatively soon after their discovery that both GalR1 and GalR2 internalize after stimulation by galanin [54,55,56,73]. After internalization, GalR1 was found to be mostly degraded by the lysosomes, while GalR2 mainly recycles to the plasma membrane [55]. The interaction of GalR1 and β-arrestin has also been corroborated recently by a PathHunter β-arrestin recruitment assay [52]. To the best of our knowledge, there are no data concerning the possible internalization of GalR3 or its association with β-arrestins.

The interaction of GalR2 and β-arrestin has been studied in more detail [53]. Both galanin and spexin are endogenous agonists of GalR2. While stimulation of the receptor by galanin induces both the Gαq- and β-arrestin–mediated pathways, spexin binding to the receptor activates only the Gαq-mediated pathway. In contrast to galanin, spexin induced minimal internalization, indicating that this endogenous peptide is a biased agonist for GalR2. Galanin and spexin were found to induce different active conformations of the receptor [53]. Spexin stabilizes a receptor conformation in which the agonist dissociates easily. In contrast, the galanin-induced active conformation of GalR2 allows retention of the agonist for a longer time, resulting in the stimulation of both Gαq- and β-arrestin–mediated pathways [53]. For GalR2, it was suggested that the ligand residence time—the dissociation rate of the agonist—plays a decisive role in internalization [53].

Although the galanin- and spexin-bound GalR2 structures have been determined [9], their comparison provides information about the receptor associated with a balanced and a G protein-biased ligand. There is no known β-arrestin-biased ligand for galanin receptors (GalRs). No structure has been determined for GalRs in complex with β-arrestins either.

## 5. Homo- and Heterodimerization of Galanin Receptors

Homo- and heterodimerization of GalRs have been reported, which may add a further layer of complexity to their signaling. GalRs are critically involved in allosteric signal integration in the central nervous system through their co-expression and assembly with other GPCRs as well as non-GPCRs, forming functional heteroreceptor complexes that display pharmacological and signaling properties that differ from those of the individual receptors [74]. This diverse heteroreceptor system provides the main molecular basis through which GalR subtypes (GalR1–GalR3) regulate various glia–neuronal systems, particularly those involved in emotional regulation and cardiovascular functions [75,76]. The most well-characterized example of GalR co-expression occurs within the serotonergic system. The subtype GalR1 and the 5-HT_1_A serotonin receptor can form heterodimers that show pronounced antagonistic receptor–receptor interactions, resulting in trans-inhibition of single-receptor signaling [77,78]. This mechanism is particularly important for controlling the main serotonin pathways that ascend from the midbrain raphe [79]. Dysfunction of these GalR–5-HT_1_A complexes in the raphe–hippocampal system has been closely associated with major depressive and anxiety disorders, making them promising new targets for antidepressant treatments [80].

A more complex arrangement, the GalR1–GalR2–5-HT_1_A heterotrimer, has been proposed to explain how different galanin fragments selectively modulate 5-HT_1_A receptors. In this model, the galanin fragment (1–15) can antagonize 5-HT_1_A signaling, whereas the full galanin peptide does not have this antagonistic effect [76,79,81]. Furthermore, galanin receptors can form isoreceptor heteromers, such as the GalR1–GalR2 heterodimer, which acts as a preferential receptor for the galanin (1–15) fragment. This structural change significantly alters ligand affinity and recognition compared to the monomeric receptors (GalR1 or GalR2) [76,82]. In addition to neurotransmitter systems, the GalR1 receptor can also form a functional heterotetramer with the μOR (μOR–GalR1), particularly in the ventral tegmental area [83]. This heteromerization induces a functional change in GalR1 signaling, redirecting it from inhibitory Gαi coupling to activation of stimulatory Gαs protein [84]. This Gαs/Gαi configuration forms an intrinsic antagonism at the level of adenylyl cyclase explaining how galanin can reduce the rewarding and addictive effects of opioid stimulation [85].

Other co-expressed complexes include GalR–α2 adrenoreceptor and GalR–neuropeptide Y receptor type 1 (GalR–NPYY1R) heteromers, both involved in mood and cardiovascular regulation [76]. Galanin can antagonize α2-adrenoreceptor signaling via a GalR–α2 heteromer, while in GalR–NPYY1R heteromers, galanin modulates NPYY1 receptor function by reducing agonist affinity, as shown in the hypothalamus [86,87].

Additionally, dopamine–galanin heteromers (D_1_R–GalR1 and D_5_R–GalR1) have been identified in the hippocampus, where they integrate dopamine and galanin signals to modulate cholinergic neurotransmission [85]. The identification of these specific GalR heteroreceptor complexes advances the understanding of galanin’s diverse effects and offers a valuable strategy for developing highly selective and effective neuromodulatory therapies [88].

## 6. Physiological and Pathological Functions of the Galaninergic System

### 6.1. Physiological Roles of the Galaninergic System

The galaninergic system regulates diverse biological processes, including energy metabolism, feeding and addiction-related behavior, neuroprotection, learning, memory, cardiovascular and renal function, sleep regulation, and nociception. Galanin plays a crucial role in feeding behavior; a relationship between galanin and glucose levels has been supported by various studies [3]. However, while galanin suppressed insulin secretion in animal models, this effect was not observed in humans [27]. Galanin was also found to be involved in the response to injury of the nervous system. Distinct functions were suggested for LC-derived galanin, as it may function as a neuromodulator and as a trophic factor acting via GalR1 and GalR2, respectively [22].

Spexin plays a role in feeding behavior and metabolism, and it is also involved in the regulation of reproduction, nociception, anxiety, and cardiovascular and renal functions [26,89]. Spexin has various metabolic effects and is involved in the regulation of glucose metabolism, insulin secretion, and energy homeostasis [44]. GALP is involved in reproduction and energy homeostasis [90]. GMAP displays antimicrobial activity [91], but it probably exerts its activity via other receptors. Alarin also acts through unknown receptors. A recent review discusses the physiological and pathological roles of alarin in more detail [92].

### 6.2. Pathological Functions

The disturbance of the galaninergic system has been associated with several human diseases, including Alzheimer’s disease (AD), diabetes mellitus, addictive behavior, and various cancers [3]. Galanin also plays a role in the pathophysiology of clinical anxiety and depression, as supported by genome-wide association studies [93]. Several excellent reviews have been published recently on the pathological roles of galanin and its receptors in cancers, obesity, and other diseases [2,94]. Here, we briefly discuss their role in some diseases.

#### 6.2.1. Diabetes Mellitus and Obesity

Spexin has also gained considerable research interest in recent years because of its role in energy metabolism [44,95]. Spexin expression is high in adipose tissue, liver, and pancreas, and the secretion of spexin is restricted to these metabolic tissues [44]. The effects of galanin and spexin are often opposed; for example, galanin appears to be orexigenic, while spexin is anorexigenic. A negative correlation was found between spexin and obesity [26]. Lower spexin but higher plasma galanin levels were correlated with type 2 diabetes [25,26,27]. The differences in their binding affinities toward GalR2 and GalR3 and mechanisms of action might explain the observed opposing effects of galanin and spexin [36]. Nevertheless, other GPCRs have also been explored as potential targets [96,97]. Glucagon-like peptide-1 receptor agonists have gained considerable attention as effective molecules for the therapy of diabetes mellitus and obesity [98,99].

#### 6.2.2. Cardiovascular Diseases

In addition to its role in diabetes mellitus, the galaninergic system is also implicated in cardiovascular diseases [75,100]. Galanin and its receptors (GalR1, GalR2, GalR3) are expressed in cardiac tissue, where they regulate important cardiac functions, including heart rate, myocardial contractility, and the heart’s protective responses to ischemic injury [101]. However, dysregulation of the galaninergic system can contribute to cardiovascular pathology by impairing the protective and regulatory mechanisms mediated by galanin and its receptors. For instance, decreased expression or dysfunction of the GalR2 receptor causes pathological cardiac remodeling and increases susceptibility to arrhythmias [102,103]. In a study by Li et al. (2024), cardiomyocyte-specific GalR2 knockout mice exhibited increased risk of atrial fibrillation, indicating that the loss of GalR2 alters calcium homeostasis in atrial cells, leading to the development of arrhythmias [103]. Similarly, impaired galanin signaling can exacerbate ischemia/reperfusion injury. Boal et al. (2022) demonstrated that reduced GalR-mediated signaling increases mitochondrial reactive oxygen species production, causing oxidative stress, hypertrophy, and fibrosis in the myocardium after ischemic insult [75,104]. This oxidative damage contributes to decreased cardiac function, highlighting the critical cardioprotective role of the galaninergic system [105]. Overall, the galaninergic system is a key regulator of cardiovascular homeostasis and plays an important role in heart disease. Continued research into this signaling pathway may lead to new treatments for heart failure, arrhythmias, and ischemic injury, ultimately improving cardiovascular outcomes.

#### 6.2.3. Alzheimer’s Disease

**Figure 6:**
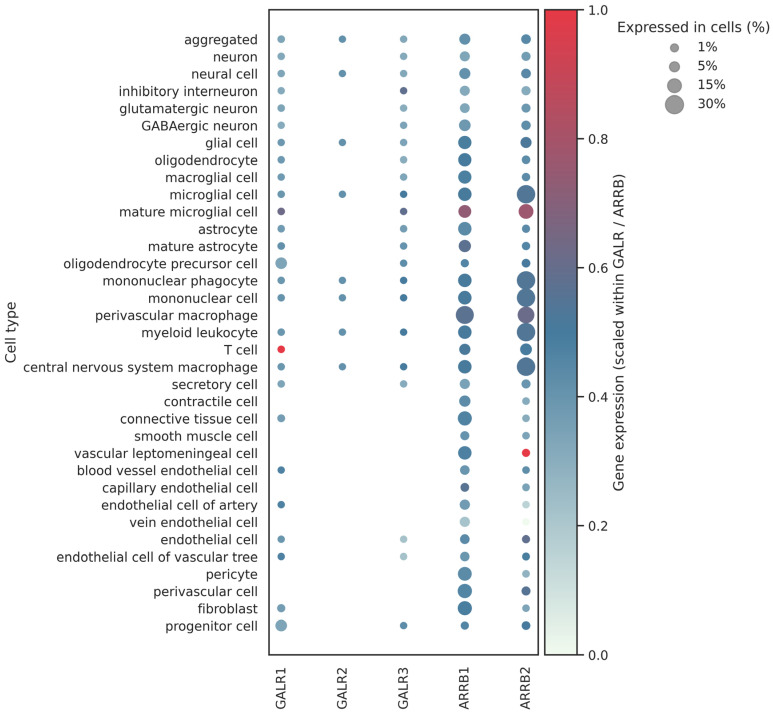
Distribution of human galanin receptors, β-arrestin1 (ARRB1) and β-arrestin2 (ARRB2) in the brain of AD patients. For each gene panel, expression values were rescaled independently, and color intensity reflects relative expression within that panel. Dot color shows globally scaled mean gene expression; dot size represents the percentage of cells expressing each gene in a given cell type. White denotes no detectable expression. Data are based on the AD brain single-cell RNA-seq dataset (https://cellxgene.cziscience.com/gene-expression, accessed on 17 November 2025). As the dataset integrates expression data from multiple sources, some cell-type labels partially overlap [113].

Altered galanin levels have also been reported in AD. AD is the most common neurodegenerative disorder, characterized by intracellular neurofibrillary tangles of phosphorylated tau and extracellular β-amyloid plaques, as well as degeneration of cholinergic neurons and progressive cognitive decline [106,107]. Currently, there is no effective treatment for AD. One of the early observations was a twofold increase in galanin receptor binding sites in various areas of post-mortem brains of AD patients; for example, in the hippocampus and the substantia nigra [108]. The levels of galanin and galanin receptors were found to be upregulated in AD patients (Figure 6) [107,109]. In post-mortem brain samples, galaninergic hyperinnervation was observed in cholinergic neurons in the basal forebrain, which was associated with a neuroprotective gene expression profile [110]. Galanin exhibited neuroprotective effects and attenuated memory impairment in some AD models, likely via GalR2- and GalR3-mediated pathways [111]. In a 2020 study, Alexandris and co-workers investigated galanin hyperinnervation in post-mortem brain samples of patients suffering from AD, Parkinson’s disease (PD), Dementia with Lewy Bodies (DLB), and mixed-type AD/DLB [112]. Although significant losses of cholinergic neurons and cholinergic projections were observed across all the studied diseases, upregulation of the galanin system and galaninergic hyperinnervation was not universal; it was especially infrequent in established AD and DLB, while it was more common during the transition from PD without cognitive impairment to PD dementia [112]. Galanin upregulation was proposed as an intrinsic response early in Lewy body diseases, which may serve as a neuroprotective mechanism.

β-arrestins have also been associated with AD pathogenesis [59,114]. In the human brain, β-arrestin1 is more abundant at the protein level than β-arrestin2, while β-arrestin2 is the predominant form in the hypothalamus (Figure 6). β-Arrestin1 levels were upregulated in post-mortem brain samples of AD patients, and their increased levels were found to be correlated with amyloid burden [115,116]. Overexpression of β-arrestin2 resulted in increased β-amyloid formation, while its deletion decreased AD pathology in various models [117]. β-Amyloid is produced via the cleavage of the amyloid precursor protein by secretases. β-Arrestins interact with γ-secretase and regulate γ-secretase complex assembly and activity, and thus β-amyloid formation [116,117]. The neuroprotective role of galanin may be mediated by GalR2 and GalR3 [111]. However, GalR2 stimulates both Gαq- and β-arrestin–regulated pathways, while GalR3 initiates Gαi/o-mediated signaling, in contrast to GalR2. There are currently no data regarding a possible relationship between the biased agonist spexin and AD. A more detailed insight into these complex processes may pave the way for the development of specific agonists/antagonists.

## 7. Therapeutic Potency of the Galaninergic System

Approximately one-third of the currently approved drugs act on GPCRs [118]. Biased agonists are of special interest because they are promising therapeutic agents with reduced side effects compared with balanced agonists. Given the multifaceted roles of β-arrestins in cellular signaling [119], many studies seek to selectively activate β-arrestin–mediated pathways. AT_1_R antagonists are often used as antihypertensive drugs; however, these molecules inhibit both G protein-dependent and β-arrestin–regulated signaling pathways. In contrast, β-arrestin–biased AT_1_R agonists are promising molecules for the therapy of acute heart failure [120]. For example, the β-arrestin–biased peptide TRV120027 (TRV027) can still stimulate multiple kinase pathways and reduce blood pressure, similar to AT_1_R blockers. Importantly, unlike traditional agents, TRV027 also offers additional benefits, such as enhancing cardiac function by boosting contractility and preserving stroke volume [121]. Despite these advantages, TRV027 did not demonstrate clinical benefit in the phase IIb BLAST-AHF trial, failing to improve 30-day outcomes compared with placebo, ultimately leading to the discontinuation of its development [122].

Clinically approved examples of biased agonists include the μOR agonist oliceridine (TRV130), which exhibits robust G protein activation but weak β-arrestin recruitment [123]. In rodents and humans, oliceridine produces potent analgesia while, at morphine-equivalent doses, causing fewer gastrointestinal and respiratory side effects. Nonetheless, accurately determining the extent of ligand bias remains challenging, and oliceridine is now considered a partial agonist [124]. A similar shift in understanding has also occurred regarding the β-arrestin–biased AT_1_R agonist TRV027, which has since been shown to activate Gαi and Gα12 proteins under certain conditions. On the other hand, Gαq-biased peptide agonists, such as TRV120055 and TRV120056, have already been developed for AT_1_R [125]. The main advantage of these ligands is that they enable the selective investigation of Gαq/11-mediated AT_1_R signaling pathways, such as fibrosis, vasoconstriction, or catecholamine release [126,127].

Galanin receptors provide a promising target for pharmacological intervention. Several agonists and antagonists are available for galanin receptors; however, their receptor subtype specificity is either limited or their binding to GalR3 has not been tested ([2,3] and references therein). The majority are peptidergic ligands derived from the sequence of the endogenous peptide galanin. Chimeric and modified peptide agonists and antagonists are also available, such as M1145 [(RG)(2)-N-galanin(2-13)-VL-(P)(3)-(AL)(2)-A-amide], a GalR2-specific agonist [128]. Taking into account the importance of Gly1 of galanin to GalR1, the galanin fragment galanin (2–11) is often used as a non-GalR1 ligand. A few nonpeptide ligands have been identified, such as galnon [7-(9-fluorenyl-methoxycarbonyl) cyclohexylalanyllysyl-amino-4-methylcoumarin], but its affinity is too low. Two 3-arylimino-2-indolone compounds (SNAP 37889 (1-phenyl-3-[[3-(trifluoromethyl)phenyl]imino]-1H-indol-2-one) and SNAP 398299 (1-[3-(2-(pyrrolidin-1-yl)ethoxy)phenyl]-3-(3-(trifluoromethyl)phenylimino)indolin-2-one) are specific GalR3 antagonists with pKi values of 7.8 and 8.3, respectively [129]. SG2A is a spexin-based, specific GalR2 agonist, which stimulates GalR2, but not GalR3 [36].

Whether these synthetic agonists and antagonists display biased signaling has not been studied. The only exception is the spexin-based GalR2 agonist, Fmoc-dA4-dQ14, which activated the Gαq-dependent pathway and did not result in receptor internalization, similarly to spexin [53]. Interestingly, the co-administration of GalR2 agonist M1145 and a NPYY1R agonist in a rat model have been reported recently, and the combination of the two agonists enhanced spatial memory and promoted neuronal survival and differentiation in the hippocampus [130]. This finding may provide a promising strategy for the treatment of cognitive decline.

Endogenous agonists may serve as biomarkers in a variety of diseases. A selection of recent studies investigating galanin and spexin as biomarkers are shown (Table 2). Using “galanin” or “spexin” as a search term, 9 clinical trials related to various diseases were identified (https://clinicaltrials.gov/search?term=galanin; accessed 15 November 2025) (Table 3).

## 8. Conclusions, Challenges and Future Perspectives

Some of the challenges are not specific to the galaninergic system but are characteristic of other GPCRs as well. The specificity of the available antibodies toward the receptor subtypes has been questioned. There is also a great need for further subtype-selective agonists and antagonists. Most of the data on the roles of galanin receptors and peptides were obtained from experiments using rats or mice. The considerable differences among species concerning receptor distribution, as well as the relative heterogeneity of neurotransmitter molecules, should be taken into account.

The least characterized human receptor is GalR3, which can be stimulated by both galanin and spexin. Moreover, spexin displays higher potency toward GalR3 than galanin. One can speculate whether spexin is also a biased agonist for GalR3, or whether it can stimulate β-arrestin-dependent signaling pathways. The fact that spexin is a biased agonist that initiates the Gαq-dependent pathway without inducing receptor internalization also raises interesting possibilities. It may help to explain how spexin is beneficial in mood disorders and other diseases. Furthermore, it may aid in the development of new GalR2 agonists that could induce biased signaling with fewer side effects in vivo. It may also provide an explanation for the observation that the effects of galanin and spexin are often opposed. Nevertheless, their distinct preferences toward GalR2 and GalR3 should also be taken into consideration, especially since GalR2 and GalR3 mediate Gαq- and Gαi/o-dependent signaling pathways, respectively.

A better understanding of the complex interplay among signaling molecules, galanin receptors, and the various signaling pathways is crucial for the future development of specific agonists with therapeutic potential.

## Figures and Tables

**Table 2:** Galanin and spexin as possible biomarkers.

Disease/Condition	Biomarker	Observation Related to Galanin/Spexin	Reference
Endometrial hyperplasia	Serum galanin level	↑ serum galanin level in patients	[131]
Schizophrenia	Serum levels of ischemia modified albumin, galanin, alarin, and meteorin-like protein	↓ serum galanin level in patients	[132]
Atopic dermatitis, mycosis fungoides	Transient receptor potential melastatin-2, spexin	↑ spexin in skin samples of patients	[133]
Colorectal cancer	Serum galanin	↓ serum galanin level in patients	[134]
Colorectal cancer	Expression of galanin and GALR1 by immunohistochemistry	↓ galanin intensity in stage IV	[135]
Gastric cancer	Serum levels of galanin and obestatin	↓ serum galanin level in patients	[136]
Breast cancer	Isthmin-1 and spexin by immunohistochemistry	↑ spexin immunoreactivity across all grades of breast carcinoma	[137]
Hip fracture patients at risk of cognitive decline	Serum galanin level	↑ serum galanin in the “cognitive decline” group	[138]
Autism spectrum disorder (ASD)	Serum galanin level	↑ serum galanin level in children with ASD relative to healthy children; ↑ galanin levels in children with severe ASD than in those with less severe disease	[139]
Obesity and insulin resistance (IR)	Plasma spexin level	↓ circulating spexin levels were associated with obesity and IR	[140]
Polycystic ovary syndrome (with liver steatosis)	Spexin level	↓ spexin level in patients strong negative correlation between spexin levels and liver steatosis grading	[141]

↓: decrease, ↑: increase.

**Table 3:** Galanin- and spexin-related clinical trials.

NCT Number	Related Disease	Peptide
NCT05446025	Hyperemesis gravidarum	Galanin
NCT00663247	Diverticular disease	Galanin
NCT02019654	Traumatic brain injury	Galanin
NCT03230149	Fabry disease (FD)	Galanin
NCT05579964	Tetralogy of fallot	Galanin
NCT01655979	Medium-term bedrest whey protein (MEP)	Galanin
NCT04514757	Postoperative atrial fibrillation (POAF)	Galanin
NCT05300802	Cyanotic congenital heart disease (CCHD)	Galanin
NCT06333548	Irritable bowel syndrome with diarrhea (IBS-D)	Spexin

## Data Availability

No new data were created or analyzed in this study.

## Abbreviations

Alzheimer’s disease (AD); angiotensin II (AngII), arginine-vasopressin (AVP); β_2_-adrenergic receptor (β_2_AR); cyclic adenosine 3,5-monophosphate (cAMP); cryo-electron-microscopy (cryo-EM); [D-Ala2, NMe-Phe4, Gly-ol5]-enkephalin (DAMGO); Dementia with Lewy Bodies (LBD); dorsal raphe nucleus (DRN); extracellular loop (ECL); extracellular signal-regulated kinase1/2 (ERK1/2); galanin-like peptide (GALP); galanin message-associated peptide (GMAP); galanin receptor 1 (GalR1); galanin receptor 2 (GalR2); galanin receptor 3 (GalR3); galanin receptors (GalRs); G protein-coupled receptor (GPCR); G protein-coupled receptor kinase (GRK); G protein–gated inwardly rectifying potassium channel, GIRK; 5-hydroxytryptamine (5-HT); intracellular loop (ICL); large-conductance Ca^2+^-activated K^+^ channel (BK); L-type voltage-dependent Ca^2+^ channel (VDCC); locus coeruleus (LC); mitogen-activated protein kinase (MAPK); neuropeptide Y receptor type 1 (NPYY1R); Parkinson’s disease (PD); phosphatidylinositol 3-kinases (PI3K); protein kinase A (PKA); protein kinase B (PKB); transmembrane helical domain (TMD); transmembrane helix (TM); transcripts per million (nTPM); type 1 angiotensin II receptor (AT_1_R); μ-opioid receptor (μOR); V_2_ vasopressin receptor (V_2_R).
